# Letter to the Editor Re: Diaz M., et al. *Nutrients* 2018, *10*, 1481

**DOI:** 10.3390/nu11020468

**Published:** 2019-02-23

**Authors:** Benjamín Martín Martínez, Maria José López Liñán

**Affiliations:** Unidad de Gastroenterología y Nutrición Infantil, Hospital de Terrassa, 08227-Terrassa, Barcelona, Spain; mjlopez@cst.cat

**Keywords:** fecal microbiota, protein hydrolyzed formulas, cow’s milk protein, tolerance acquisition, non-IgE mediated allergy

## Dear Editor,

We have read with interest the article published by Diaz et al. about the effects of extensively hydrolyzed cow’s milk formulas (eHF) or vegetable formulas (soy and rice) on the microbiota in children with a non-IgE mediated (NIM) cow’s milk protein allergy (CMPA) under a restrictive diet [1]. Although we believe it is a very interesting topic, we do not agree on some points and conclusions that are derived from this study, as explained below.

Guidelines and recommendations on the nutritional treatment of infants diagnosed with a mild to moderate cow’s milk protein allergy (CMPA) advise the use of eHF formulas as the first option, whereas elemental formulas based on free amino acids are recommended in case of failure of the eHF formulas. Soy formulas (SF) are an option in CMPA infants older than six months, due to the presence of phytoestrogens.

In recent years, hydrolyzed rice protein formulas (HRFs) have been included in the protocols and guidelines as a first option in the treatment of CMPA, as rice is one of the less allergenic foods (<1%) and does not contain phytoestrogens [2,3]. Moreover, HRFs have a better palatability and are a good option in children who reject the eHF formulas or in vegetarian or vegan families, with efficacy and nutritional safety similar to eHF [4]. Therefore, according to the latest published recommendations on CMPA, HRFs are a first line option in infants with CMPA from the first day of life, at the same level of eHF, in those countries where HRFs are available. 

The research topic in the study of Diaz et al. is interesting, but the number of infants both in the study group (n = 17), as well as in the control group of healthy children of same sex and age (n = 10), is too low. Moreover, if we look at the number of infants in each formula group, the number of cases studied is even lower: n = 12 in the eHF group, n = 2 in the SF group and n = 3 in the HRF group. 

Since this is a cohort study, it is assumed that infants were not randomly assigned to each formula, and we think this is an important limitation for the interpretation of the results of the study, particularly with regard to causality between the type of formula ingested, the observed pattern of the microbiota and tolerance acquisition to cow’s milk proteins. The authors neither explain what the criteria to allocate the infants to the eHF were, nor what the vegetable (soy or hydrolyzed rice) formula groups were.

Secondly, the authors do not provide enough information about the diet of the infants before their inclusion in the study. The study was carried out in infants with non IgE mediated CMPA with ages between 13 and 23 months, with a mean age of 17 months. Considering that diagnosis of NIM-CMPA is usually performed at ages below six months, the authors should provide information about the type of diet they received from diagnosis until the beginning of the study. We think this is a key factor because it can clearly influence the pattern of the microbiota in these infants, and this factor could explain, at least in part, the observed differences in the acquisition of tolerance between infants in the eHF, SF, and HRF groups; that is, none of the three infants who were fed with HRF acquired tolerance, whereas the infants fed eHF and SF acquired tolerance by the end of the study. Another important difference between controls and infants with NIM-CMPA is that the latter followed a diet free of dairy products for six months. Indeed, this can be a factor modifying the microbiota (e.g., fermented dairy products are an important source of lactobacillus at this age).

Another important missing piece of information in the article is related to the type of formula eHF used in the study. The authors do not provide information about the type of milk hydrolysate used in the eHF group (casein or whey proteins), and whether they were lactose free or with added lactose (with prebiotic effect of undigested lactose). All these factors may have a strong impact on the microbiota of the children.

The third point is related to the analysis of microbiota composition in the infants. Diaz et al. find that those infants who do not become tolerant in the study (those fed HRF) present less abundance of *Coriobacteriaceae* and *Bifidobacteriaceae* than those who become tolerant. However, they also mention that infants fed vegetal protein formulas, both rice and soy, have less *Coriobacteriaceae* than those fed eHF formulas. They do not provide any hypothesis to the fact that infants fed SF with lower *Coriobacteriacea* levels become tolerant (unfortunately, they do not provide details on the levels per feeding group). Also, the authors do not explain the fact that *Coriobacteriaceae* are also less abundant in healthy controls and vegetal formula in comparison with NIM-CMPA infants. Ultimately, the lack of data on diet composition in the different feeding groups, as well as the lack of longitudinal data on microbiota composition, makes it difficult to interpret or make conclusions about the microbiota results in this study. 

Finally, the authors suggest in the discussion of the article that the use of vegetable formulas may impair the acquisition of tolerance due to the absence of exposure to immunomodulatory peptides. We believe that this conclusion cannot be drawn from the results of the study from Diaz et al., given the small number of infants in each study group, as well as the other confounding factors, mentioned in the previous paragraphs, that were not controlled in the present study and may have influenced the obtained results. The role of immunomodulatory peptides in the acquisition of tolerance was suggested in the study performed by Canani et al., which is a non-randomized study, with an eHF formula with LGG (*Lactobacillus Rhamnosus* GG) [5,6].

Contrary to the suggestion of the authors, several clinical trials in infants have found similar rates for the acquisition of tolerance between infants with CMPA fed extensively hydrolyzed cow’s milk protein formulas and hydrolyzed rice formulas [7], besides being effective for the treatment of CMPA [7,8]. Moreover, a study published by Terracciano et al. in 2009 found that infants and children with CMPA who received hydrolyzed rice or a soy-based formula for the dietary management of their condition achieved tolerance earlier than their peers on an extensively hydrolyzed cow’s milk formula. This fact led to the consideration that the elimination from the diet of any cow’s milk protein residue may accelerate the induction of tolerance and would be adequate for the management of CMPA [9].
